# Isolation and characterization of 15 SSR loci for the endangered European tetraploid species *Gladiolus palustris* (Iridaceae)

**DOI:** 10.1002/aps3.1245

**Published:** 2019-05-08

**Authors:** Tamás Malkócs, Shyryn Almerekova, Judit Bereczki, Judit Cservenka, Emese Meglécz, Gábor Sramkó

**Affiliations:** ^1^ Department of Botany University of Debrecen Debrecen Hungary; ^2^ Department of Biodiversity and Bioresources Al‐Farabi Kazakh National University Almaty Kazakhstan; ^3^ Department of Evolutionary Zoology and Human Biology University of Debrecen Debrecen Hungary; ^4^ Balaton Uplands National Park Directorate Csopak Hungary; ^5^ Aix Marseille Univ, Avignon Université, CNRS, IRD, IMBE Marseille France; ^6^ MTA‐DE “Lendület” Evolutionary Phylogenomics Research Group Debrecen University of Debrecen Hungary

**Keywords:** conservation, *Gladiolus palustris*, Habitats Directive, microsatellite, polyploid, population genetics

## Abstract

**Premise:**

*Gladiolus palustris* (Iridaceae) is an endangered European perennial tetraploid herb with special conservation interest in the European Union. Microsatellite markers can serve as effective tools for the conservation genetics of this species.

**Methods and Results:**

We utilized a 454 pyrosequencing approach to identify simple sequence repeat (SSR) regions in a microsatellite‐enriched library. Of all SSR regions, 46 were screened for specific PCR amplification, and 15 were found to be applicable in the target species. We found 1.62–3.08 alleles per population (effective alleles: 1.58–2.08) that indicated moderate to high genetic diversity values (0.28–0.44) in three pilot populations. Cross‐species amplification was less effective in *G. imbricatus* and *G. tenuis*.

**Conclusions:**

The primers reported here can be used for the population genetic characterization of *G. palustris*. They will help us to better understand the conservation genetics of this highly endangered species.

The genus *Gladiolus* L. comprises approximately 260 species confined to the Old World, which are mainly found on the African continent. Only seven species are native to Europe, with the highest diversity in the Mediterranean (Valente et al., 2011), but some extend farther to the north and occur in temperate regions. One of these species is *G. palustris* Gaudich., an endemic species of Central Europe that occurs from eastern France to Hungary, and from southern Germany to northern Italy and the northern Balkans (Szczepaniak et al., 2016). It has a reported chromosome number of 2*n* = 60, which corresponds to the tetraploid level in this genus (Hamilton, 1980). Because this species lives in a highly endangered habitat, the ecotone between wet meadows and dry grasslands, it is declining throughout its distribution (Schnittler and Günther, 1999) and is included in Annex II of the Habitats Directive of the European Union (Council Directive 92/43/EEC; Council of the European Union, 1992). It is included on several national red lists (National Red List Project [https://www.nationalredlist.org/]) and is incorporated in the International Union for Conservation of Nature (IUCN) Red List as data deficient (Bilz, 2011). For the effective conservation of *G. palustris*, it is necessary to develop genetic markers providing resolution at the population level.

Here, we report the development and characterization of 15 microsatellite markers to investigate the conservation genetics of *G. palustris*. We also tested the cross‐species utility of 11 polymorphic loci in two related European species, *G. imbricatus* L. and *G. tenuis* M. Bieb. The former species forms hybrids with *G. palustris*, which indicates the close genetic relationship between the two species (Szczepaniak et al., 2016). *Gladiolus tenuis* is also included as an Eastern European relative of *G. imbricatus* (Gabrielian, 2001). Previous genetic studies of *G. palustris*, which are scarce, have utilized commonly used plastid markers and amplified fragment length polymorphism (AFLP) analysis (Szczepaniak et al., 2016). The markers evaluated here are expected to provide a fundamental tool for population genetic studies for the purpose of conservation and management of this species with high conservation importance.

## METHODS AND RESULTS

Leaves were dried in silica gel, and genomic DNA was extracted using a modified cetyltrimethylammonium bromide (CTAB) protocol (Doyle and Doyle, 1987) described in detail elsewhere (Sramkó et al., 2014). Microsatellite isolation followed a protocol based on 454 sequencing (Sinama et al., 2011), in which DNA of one plant from five geographically distant populations (Appendix 1) was mixed equimolarly and enriched for microsatellites using the following probes: (TG)_10_, (TC)_10_, (AAC)_8_, (AAG)_8_, (AGG)_8_, (ACG)_10_, (ACAT)_6_, and (ACTC)_6_. The purified and enriched library was used in the 454 GS‐FLX Titanium library preparation (Roche Applied Science, Penzberg, Germany) following the manufacturer's protocols performed by Genoscreen Ltd. (Lille, France). The software QDD version 3.1.2 (Meglécz et al., 2014) was used for the selection of sequences for primer design using default parameters. For contamination detection, sequences were compared with the nucleotide database of the National Center for Biotechnology Information (NCBI; downloaded in July 2015) using BLAST. Sequences were screened for transposable elements using the RepeatMasker Libraries made available by the Genetic Information Research Institute (GIRI; http://www.girinst.org [accessed February 2015]). Primer pairs were selected from the QDD output according to the following criteria: (1) only pure microsatellites were allowed in the target region with at least six repeats, (2) repeats of (AT)_n_ were excluded, (3) the primer alignment score to the amplified sequence had to be lower than 6, (4) primers had to be at least 10 bases away from the microsatellite motif, and (5) consensus sequences were discarded. Out of the 110 potential primer pairs, 46 loci were selected for screening based on fragment length and melting temperature (*T*_m_) of their forward and reverse primers. All loci were selected based on the criterion that primer annealing temperature (*T*_a_) had to be 64°C.

Initial testing of the 46 primers for specific PCR amplification was based on a single individual from the Nyirád population (Appendix 1), and resulting PCR products were evaluated by electrophoresis on chilled 2% agarose gel. The PCR mixture contained: 2× DreamTaq Green Buffer, 0.2 mM dNTP (each), 1 mg/mL bovine serum albumin, 0.5 μM of each primer, and 0.05 units DreamTaq Green DNA Polymerase in a final volume of 10 μL (all PCR reagents were purchased from Thermo Fisher Scientific, Carlsbad, California, USA). PCR cycling conditions were: denaturation at 95°C for 2 min; 40 cycles of 15 s at 95°C, 30 s at 62°C, and 1 min at 72°C; with a final extension step at 72°C for 10 min. All PCRs were run using the above conditions. Twenty‐five loci presented specific products. These were tested in a sample size of five plants from the Raposka population (Appendix 1), searching for potentially polymorphic loci using the same electrophoretic conditions as above. Fifteen loci were selected for further analyses based on apparent length differences between the five samples as assessed visually. Forward primers of the selected loci were labeled with a fluorophore dye at their 5′ end (Table 1), and the SSR loci were PCR amplified in three Hungarian populations of our target species represented by 25, 20, and 12 individuals (Table 2, Appendix 1). The fluorescent‐labeled PCR products were run on an ABI 3130 Genetic Analyzer (Applied Biosystems, Foster City, California, USA). The products were multiplexed according to their predicted fragment length and fluorescent label type, containing varying amounts of PCR product per locus (1.5–4 μL) based on band intensity (Appendix S1). One microliter of the multiplexed PCR products was added to 0.25 μL of GeneScan 500 LIZ Size Standard (Applied Biosystems) and 14.75 μL of Hi‐Di formamide (genetic analysis grade; Applied Biosystems) before loading on the Genetic Analyzer. Genotype calling was carried out manually using PeakScanner version 1.0 (Applied Biosystems). Because of the tetraploid nature of our target species, genotypes were evaluated and analyzed using GenoDive version 2.0b27 (Meirmans and Van Tienderen, 2004) as this software can take allele copy number ambiguity into account in partial heterozygotes.

**Table 1:** Characteristics of 15 microsatellite loci developed in *Gladiolus palustris*

Locus[Link]	Primer sequences (5′–3′)	Repeat motif	Allele size range (bp)	Fluorescent dye	GenBank accession no.
GlPal01[Link]	F: TTCTGCAAAGCTAACAGGCG	(CT)_6_	201	PET	MG266909
	R: CCACTGCCATTATCGGTCGA				
GlPal03[Link]	F: TTGTTACTGGTGCGGACTCC	(CT)_9_	211	NED	MG266910
	R: CAGGTCCGATTGCTTGAGGA				
GlPal04[Link]	F: TTTGGGACGGGTATGCGTAG	(GA)_9_	137	6‐FAM	MG266911
	R: TTCGCTTTCGTGGAAGTCCA				
GlPal08	F: ATGCCTTTGTCCTCTCACCT	(CT)_8_	137–141	VIC	MG266912
	R: TTTGTCCCTAATTGGAACACGTC				
GlPal11	F: CGGGAATAGCAACTACCCGG	(AG)_6_	136–138	PET	MG266913
	R: GTTGCCCTTGCGGTTGATC				
GlPal13	F: ATTGCCAAGGAGGTACAGGC	(GAA)_6_	95–107	6‐FAM	MG266914
	R: GCAAACCCACTCTGCTTCCT				
GlPal14	F: CCAAGTAAGTGATGGCGGC	(GAG)_8_	189–207	6‐FAM	MG266915
	R: GGGTCTAGAGAAGGCTTGGG				
GlPal21	F: GTGGGTTCCAAGCTTGCATG	(GA)_16_	122–142	NED	MG266916
	R: TCATTGGCCGCGACAGAG				
GlPal22	F: TGAAACCCTACACGTAACCCT	(AG)_9_	293–305	6‐FAM	MG266917
	R: ATGTAGGGTGATGGTGGCTG				
GlPal24	F: ACATTCCTATTTGGACGGTCCA	(TC)_8_	291–297	VIC	MG266918
	R: AGTTCCCTAGTCATCTATGGCT				
GlPal37	F: ACGTGAGTGATCAATTAAAGGGC	(AAC)_6_	261–270	NED	MG266919
	R: CAACAGCACTGTGAGCACC				
GlPal39	F: ATCCCTTTCCTAATTCCTTCCC	(AG)_8_	238–240	6‐FAM	MG266920
	R: TGATGAAGAGGATTCCGACGAC				
GlPal41[Link]	F: AAACCCTCACTTCGGAGATCA	(GA)_7_	287	PET	MG266921
	R: TAAAGTCAGTCAGCTGTAACACTG				
GlPal42	F: TCGGAAGGTAACAGAGGGAAC	(AAG)_6_	242–251	VIC	MG266922
	R: GTCGAAACAGAGGAGATCGGG				
GlPal46	F: GGGTCATCGCCTGTCATGAA	(GAA)_8_	207–219	VIC	MG266923
	R: TCGTATCGGCTTGTTGGCTG				

Annealing temperature (*T*_a_) for all primers is 64°C.

Monomorphic in the test sample set.

**Table 2:** Genetic diversity of 11 polymorphic microsatellite markers in three populations of *Gladiolus palustris* as calculated in GenoDive version 2.0b27.[Link]

Locus	Raposka (*n* = 25)			Nyirád (*n* = 20)			Bátonyterenye (*n* = 12)		
	*A*	*A*_e_	*H*_e_	*A*	*A*_e_	*H*_e_	*A*	*A*_e_	*H*_e_
GlPal08	3	2.048	0.522	2	2.000	0.513	2	2.000	0.522
GlPal11	2	1.992	0.508	2	2.000	0.513	2	2.000	0.522
GlPal13	2	1.986	0.512	2	1.978	0.512	1	1.000	0.000
GlPal14	5	2.183	0.557	4	2.442	0.609	2	1.578	0.387
GlPal21	4	3.041	0.682	4	3.221	0.702	2	2.000	0.522
GlPal22	5	2.405	0.601	6	3.058	0.694	1	1.000	0.000
GlPal24	3	1.162	0.144	4	1.223	0.190	1	1.000	0.000
GlPal37	4	3.550	0.727	4	3.849	0.751	3	3.000	0.686
GlPal39	2	1.683	0.418	2	1.090	0.087	1	1.000	0.000
GlPal42	3	2.100	0.534	4	2.164	0.551	2	2.000	0.522
GlPal46	5	2.026	0.521	4	2.048	0.526	2	1.912	0.502
Overall	3.077	2.014	0.440	3.077	2.083	0.434	1.615	1.576	0.282

Note

*A* = number of alleles; *A*_e_ = effective number of alleles; *H*_e_ = expected heterozygosity; *n* = number of individuals.

Locality and voucher information are provided in Appendix 1.

Although we expected all 15 loci to be variable based on visual inspection, only 11 loci proved to be variable. We report population sizes, number of alleles, effective number of alleles, and levels of expected heterozygosity (Table 2). We do not report observed heterozygosities, as these calculations are not based on the corrected allele frequencies and are therefore biased (Meirmans and Van Tienderen, 2004). The number of alleles and effective number of alleles ranged from one to 6 and 1.000 to 3.849 per locus in the three studied populations, respectively. The mean level of expected heterozygosity was 0.385 (0.000–0.751) (Table 2). Cross‐amplification was tested on the loci found to be polymorphic in the focal species in five individuals of *G. imbricatus* and three individuals of *G. tenuis* from various locations across their distribution (Appendix 1) using the same PCR conditions as described above; only four of the 11 polymorphic loci were amplified (Table 3). This may be caused by the relatively distant phylogenetic relationship between our target species and the species of the *G. imbricatus* group.

**Table 3:** Cross‐amplification of 11 polymorphic microsatellite loci developed for *Gladiolus palustris* in the related taxa *G. imbricatus* and *G. tenuis*.[Link]

Locus	*Gladiolus imbricatus* (*n* = 5)	*Gladiolus tenuis* (*n* = 3)
GlPal08	+	+
GlPal11	–	–
GlPal13	–	–
GlPal14	+	+
GlPal21	–	–
GlPal22	+	+
GlPal24	–	–
GlPal37	+	+
GlPal39	–	–
GlPal42	–	–
GlPal46	–	–

Note

+ = successful amplification; – = unsuccessful amplification; *n* = number of individuals.

Locality and voucher information are provided in Appendix 1.

## CONCLUSIONS

We identified the first polymorphic microsatellite markers in *G. palustris*. These 11 polymorphic markers are crucial in population genetic studies in the species, providing information on genetic diversity, levels of inbreeding, and gene flow. Thus, they present a much‐needed tool in the conservation and management of this critically endangered species.

## Supporting information


**APPENDIX S1.** Multiplexing groups and amounts of PCR product added to the multiplex mix for each locus.

## Data Availability

Sequence information for the developed primers has been deposited to the National Center for Biotechnology Information (NCBI); GenBank accession numbers are provided in Table 1.

## APPENDIX 1 Geographic and voucher information of *Gladiolus* populations represented in this study.

**Table:**

Species	Location, ISO country code	*n*	Geographic coordinates	Voucher no.^a^
*Gladiolus tenuis* M. Bieb.	Horodyshche, UA	1	49°02′19.4″N, 39°39′32.7″E	DE‐Soo‐45670
*Gladiolus tenuis*	Krasny Kurgan, RU	1	43°58′19.8″N, 42°35′54.8″E	DE‐Soo‐45671
*Gladiolus tenuis*	Mikhaylovka, RU	1	49°50′19.3″N, 43°07′48.9″E	DE‐Soo‐45672
*Gladiolus imbricatus* L.	Prejmer, RO	1	45°43′36.8″N, 25°44′04.5″E	DE‐Soo‐45681
*Gladiolus imbricatus*	Lunca de Jos, RO	1	46°34′18.2″N, 25°59′22.9″E	DE‐Soo‐45679
*Gladiolus imbricatus*	Zakopane, PL	1	49°16′40.7″N, 19°54′11.0″E	DE‐Soo‐45682
*Gladiolus imbricatus*	Komlóska‐Újhuta, Zsidó‐rét, HU	1	48°21′58.8″N, 21°28′23.0″E	DE‐Soo‐09423
*Gladiolus imbricatus*	Cluj‐Napoca, Valea morilor, RO	1	46°41′51.5″N, 23°35′56.4″E	DE‐Soo‐38677
*Gladiolus palustris* Gaudich.^b^^,^ c	Raposka, HU	25	46°51′39.9″N, 17°25′01.3″E	DE‐Soo‐45673
*Gladiolus palustris* d	Nyirád, HU	20	47°00′02.1″N, 17°25′31.5″E	DE‐Soo‐45674
*Gladiolus palustris* c	Bátonyterenye, Lengyendi‐Galya, HU	12	47°55′05.8″N, 19°54′12.8″E	DE‐Soo‐09419
*Gladiolus palustris* c	Ásotthalom, Csodarét, HU	1	46°11′55.0″N, 19°49′48.1″E	DE‐Soo‐45683
*Gladiolus palustris* c	Schwangau, DE	1	47°33′38.5″N, 10°43′53.7″E	DE‐Soo‐45684
*Gladiolus palustris* c	Augsburg, DE	1	48°18′24.3″N, 10°55′43.2″E	DE‐Soo‐45685

Note

ISO = International Organization for Standardization; *n* = number of individuals included in this study.

a Voucher specimens are deposited at the herbarium of the University of Debrecen (DE), Debrecen, Hungary.

b Five individuals from this population were included in searching for potentially polymorphic loci during marker evaluation.

c DNA of a single individual from this population was used for the construction of the 454 sequencing library.

d This individual was used for the initial test of specific PCR amplification of 46 primers.
