# Supplementary material for: Isolation and characterization of 15 SSR loci for the endangered European tetraploid species Gladiolus palustris (Iridaceae)
Source: Appl Plant Sci. 2019 May 8;7(5):e01245. doi: 10.1002/aps3.1245 (PMC6526657; doi:10.1002/aps3.1245)
Supplement: Supplementary file 1 — APPENDIX S1. Multiplexing groups and amounts of PCR product added to the multiplex mix for each locus. [file APS3-7-e01245-s001.docx]

**APPENDIX S1**. Multiplexing groups and amounts of PCR product added to the multiplex mix for each locus.^a^

Multiplex 1:

| **Locus** | **Fluorescent label type** | **Allele size (bp)** | **Volume added (µL)** |
| --- | --- | --- | --- |
| GlPal03 | NED | 211 | 3.50 |
| GlPal04 | 6-FAM | 137 | 2.50 |
| GlPal13 | 6-FAM | 95–107 | 1.50 |
| GlPal14 | 6-FAM | 189–207 | 2.00 |
| GlPal21 | NED | 122–142 | 2.50 |
| GlPal22 | 6-FAM | 293–305 | 3.00 |
| GlPal41 | PET | 287 | 2.00 |
| GlPal42 | VIC | 242–251 | 1.50 |

Multiplex 2:

| **Locus** | **Fluorescent label type** | **Allele size (bp)** | **Volume added (µL)** |
| --- | --- | --- | --- |
| GlPal01 | PET | 201 | 1.50 |
| GlPal08 | VIC | 137–141 | 2.00 |
| GlPal11 | PET | 136–138 | 2.50 |
| GlPal24 | VIC | 291–297 | 4.00 |
| GlPal37 | NED | 261–270 | 2.00 |
| GlPal39 | 6-FAM | 238–240 | 3.00 |
| GlPal46 | VIC | 207–219 | 2.00 |

^a^PCR reactions per sample were first mixed according to the volumes provided, and then 1 µL of the multiplexed PCR product was loaded on the ABI 3130 Genetic Analyzer (Applied Biosystems, Foster City, California, USA).
